# Exogenous Calcium Alleviates Low Night Temperature Stress on the Photosynthetic Apparatus of Tomato Leaves

**DOI:** 10.1371/journal.pone.0097322

**Published:** 2014-05-14

**Authors:** Guoxian Zhang, Yufeng Liu, Yang Ni, Zhaojuan Meng, Tao Lu, Tianlai Li

**Affiliations:** 1 Horticulture Department, Shenyang Agricultural University, Shenyang, Liaoning Province, China; 2 Key Laboratory of Protected Horticulture of Ministry of Education, Shenyang, Liaoning Province, China; 3 Key Laboratory of Protected Horticulture of Liaoning Province, Shenyang, Liaoning Province, China; US Naval Reseach Laboratory, United States of America

## Abstract

The effect of exogenous CaCl_2_ on photosystem I and II (PSI and PSII) activities, cyclic electron flow (CEF), and proton motive force of tomato leaves under low night temperature (LNT) was investigated. LNT stress decreased the net photosynthetic rate (Pn), effective quantum yield of PSII [Y(II)], and photochemical quenching (qP), whereas CaCl_2_ pretreatment improved Pn, Y(II), and qP under LNT stress. LNT stress significantly increased the non-regulatory quantum yield of energy dissipation [Y(NO)], whereas CaCl_2_ alleviated this increase. Exogenous Ca^2+^ enhanced stimulation of CEF by LNT stress. Inhibition of oxidized PQ pools caused by LNT stress was alleviated by CaCl_2_ pretreatment. LNT stress reduced zeaxanthin formation and ATPase activity, but CaCl_2_ pretreatment reversed both of these effects. LNT stress caused excess formation of a proton gradient across the thylakoid membrane, whereas CaCl_2_ pretreatment decreased the said factor under LNT. Thus, our results showed that photoinhibition of LNT-stressed plants could be alleviated by CaCl_2_ pretreatment. Our findings further revealed that this alleviation was mediated in part by improvements in carbon fixation capacity, PQ pools, linear and cyclic electron transports, xanthophyll cycles, and ATPase activity.

## Introduction

Tomato (*Lycopersicon esculentum* Mill.), an important vegetable crop cultivated in Northeastern China, often encounters low night temperature (LNT) stress followed by warm sunny days. Our previous studies showed that carbon fixation capacity, linear electron transport, and active oxygen-scavenging enzymes is inhibited by LNT stress followed by growth light [1], [2]. LNT stress can induce photoinhibition, which refers to reduction in photosynthetic efficiency under excessive light [3], [4] of tomato leaves.

Photoinhibition takes place not only under high light but also under low light at low temperature [5], [6]. Generally, photosystem II (PSII) is regarded as the original site and the principal component of photoinhibition [7], [8]. Nevertheless, some studies have suggested that preferential PSI photoinhibition in plants may occur under low or moderate light intensity accompanied by low temperature [9]–[11]. The conversion of excitation energy into the energy of separated charges and water–plastoquinone oxido-reductase activity is inadvertently coupled with the formation of reactive oxygen species (ROS) [12]. Nevertheless, excess ROS accumulation causes peroxidation of thylakoid membrane lipids, degradation of D1 protein, and photoinhibition of PSII and PSI [13]–[16]. Non-photochemical quenching (NPQ) mechanism and alternative electron transport pathways can dissipate excessive excitation pressure accumulated in PSII reaction centers without causing adverse effects. Lumen acidification can activate NPQ mechanisms, including the xanthophyll cycles and the protonation of residues on key light-harvesting complex (LHC) components [17]. Aside from NPQ, cyclic electron flow (CEF) is another major protection mechanism for PSII under excessive light conditions because it generates extra proton gradient (ΔpH) across the thylakoid membrane [18]–[21]. Compared with PSII, PSI has two mechanisms protecting it from selective photoinhibition: one is blockage of linear electron transport or photoinhibiton of PSII and the other is CEF around PSI. Closure of PSII can reduce the electrons transferred to PSI. Meanwhile, CEF can oxidize P700 into P700^+^ and consume excess reducing power of NADPH [22], [23]. Researchers have strived for years to fully understand the mechanism of photoinhibition and photoprotection and to find a proper method to alleviate photoinhibition.

Some studies have shown that photoinhibition of plants under abiotic stress can be alleviated by exogenous Ca^2+^ [24]–[26]. Ca^2+^ regulates various cellular activities, including cell division and elongation, cytoplasmic streaming, photomorphogenesis, and plant defense against environmental stresses [27]. Exogenous Ca^2+^ can improve plant photosynthesis under various stresses [26], [28], [29]. It can also raise stomatal conductance, activities of key enzymes in the Calvin–Benson cycle, photosynthetic electron transport, and antioxidant capacity under stress caused by heat, cold as well as low light stress [26], [30]–[32]. Under abiotic stress, Ca^2+^-binding proteins act as stress sensors and interact with downstream effector molecules, including calmodulin (CaM), Ca^2+^-dependent protein kinases, and calcineurin B-like proteins. Ca^2+^ and CaM are constitutive and functional components of chloroplasts. Studies have shown that Ca^2+^ concentration can reach 15 mM-25 mM in chloroplasts. Most of the calcium is believed to be associated with thylakoid membranes and macromolecules within the thylakoid lumen [33]. Chloroplastic proteins, such as PsaN, NADK, and TIC32, can function properly only when bound to CaM [34]–[36]. Ca^2+^ can also regulate the cyclic photosynthetic electron transfer through a complex consisting of a calcium sensor (CAS), ANR1, and PGRL1 [37] In addition, Ca^2+^ can help maintain thylakoid membrane integrity and increase the stack of grana to promote light absorption [38].

In the present study, the effects of exogenous Ca^2+^ pretreatment on the carbon fixation capacity, PSII and PSI activities, plastoquinone (PQ) pools, thylakoid membrane, ATPase activity, ΔpH across the thylakoid membrane, and zeaxanthin formation in tomato leaves under LNT stress were investigated. Our results provide insights into the effects of Ca^2+^ on photoinhibition and photoprotection mechanisms including CEF and xanthophyll cycles of tomato leaves under LNT stress.

## Materials and Methods

### Plant Materials and Growth Conditions

Tomato seeds from a popular variety (‘Liaoyuanduoli’) grown at the Shenyang Agricultural University in Northeast China were germinated and grown in pots in a heated greenhouse with average day/night temperatures of 25°C/15°C under natural light(about 600 µmol·m^−2^·s^−1^) at a relative humidity (RH) of 60%.

### Ca^2+^ Pretreatment and Low Night Temperature Treatment

Plants were divided into four groups at the six-leaf stage, with 30 pots in each group. The first and second groups were sprayed with distilled water, whereas the third and fourth groups were sprayed with 27 mM CaCl_2_ and 10 mM ethylene glycol tetraacetic acid (EGTA), respectively. EGTA is a Ca^2+^ chelator and blocker of Ca^2+^ ionophore and Ca^2+^channels [39]. It is often used to reduce the extracellular Ca^2+^ concentration by chelation in order to eliminate the physiological effects of calcium in a variety of experiments. Each group was sprayed with 120–150 ml of the respective solution each time, twice a day for 3 d. On the third day at 18∶00, the last three groups were subjected to LNT treatment by transferring to a phytotron. The treatment was done for 12 h every day (from 18∶00 to 06∶00) for 3 d. The night period coincided with the LNT period. The environmental conditions were as follows: a 12 h photoperiod with a photosynthetic photon flux density (PPFD) of 600 µmol·m^−2^·s^−1^, 25°C/6°C (day/night), and 60% RH. Plants in the control phytotron were subjected to 25°C/15°C (day/night) and the same environmental conditions described above. After 3 d of treatment (at the onset of the light period directly after the dark period), measurements were performed on the fourth fully expanded functional leaves using four replicates from different pots.

### Measurement of Gas Exchange, Chlorophyll Fluorescence, and P700 Parameters

GFS-3000 and DUAL-PAM-100 measuring systems (Heinz Walz, Effeltrich, Germany) were used to simultaneously obtain light-dependency curves for gas exchange, chlorophyll fluorescence, and P700 redox state as described by Yamori et al.[40] with minor modifications. The net photosynthetic rate (Pn) and light-adapted fluorescence parameters were recorded after 3 min of exposure to different PPFDs (13, 37, 70, 126, 216, 339, 531, 825, 1287 µmol·m^−2^·s^−1^). All measurements were performed at a CO_2_ concentration of approximately 400±10 µmol·mol^−1^.

The following Chl fluorescence parameters were calculated: Fv’/Fm’ = (Fm’−Fo’)/Fm’, qP = (Fm’−Fs)/(Fm’−Fo’), Y(II) = (Fm’−Fs)/Fm’ [41], and Y(NO) = Fs/Fm, Y(NPQ) = 1−Y(II) − Y(NO) [42], where Fo’ is the minimum fluorescence in the light-adapted state, Fm and Fm’ (Fm was determined after an overnight dark adaptation) are the dark-adapted and light-adapted maximum fluorescence upon illumination with a pulse (300 ms) of saturating light (10000 µmol·m^−2^·s^−1^), respectively. Fs is the light-adapted steady-state fluorescence, Fv’/Fm’ is the light-adapted maximum quantum yield of PSII, qP is the photochemical quenching coefficient, Y(II) is the effective quantum yield of PSII, Y(NO) is the quantum yield of non-regulated energy dissipation of PSII, and Y(NPQ) is the quantum yield of regulated energy dissipation of PSII. Y(NO) consists of non-photochemical quenching due to photoinactivation and constitutive thermal dissipation that are very stable despite environmental stresses [43].

The quantum yield of PSI [Y(I)] is defined by the proportion of the overall P700 that is reduced in a given state and not limited by the acceptor side. It was calculated from the complementary PSI quantum yields of non-photochemical energy dissipation Y(ND) and Y(NA), i.e., Y (I) = 1–Y(ND) –Y(NA), where Y(ND) and Y(NA) are the quantum yields of non-photochemical energy dissipation in PSI due to donor and acceptor side limitations, respectively. Y(ND) = 1–P700red and Y (NA) = (Pm–Pm’)/Pm [44]. Pm, which is analogous to Fm, was determined by applying a saturation pulse after pre-illumination with far-red light. It represents the level at P700 is fully oxidized. Pm’,?was also defined in the same way as the fluorescence parameter Fm’. P700red, which was determined in a given state with the help of a saturation pulse, represents the fraction of overall P700 that is reduced in a given state.

It has recently been noted that Y(II) and Y(I) may represent data from different parts of leaf tissues [5]. The Chl fluorescence signal is mainly measured from leaf mesophyll near the leaf surface, while the P700 signal comes from the whole tissue. As a result, LEF is possibly underestimated and consequently CEF would be overestimated. Even though there might be some overestimation of CEF, we believe that the trend of change in the ratio of CEF to LEF is reliable. Therefore, we used the parameter, Y(CEF)/Y(II), to estimate the extent of CEF as described in a previous study [5]. Y(CEF)/Y(II) = [Y (I) –Y(II)]/Y(II).

### Measurement of Fast Chl a Fluorescence Induction Kinetics Curves and PQ Pools

The fast induction kinetics of Chl a fluorescence was monitored using automated routines provided by the Dual-PAM software as described by Schreiber [45]. The rapid induction kinetics upon onset of strong continuous illumination was investigated on the tomato leaves after dark adaptation for 20 min. The states of donor and acceptor sides of PS II is reflected by a log timescale assessment of the relative variable fluorescence (Vt) [46]. Vt is defined as the ratio of variable fluorescence to the maximal variable fluorescence F_V_ (Fm-Fo), i.e., Vt = (Ft-Fo)/(Fm-Fo), where Ft is the fluorescence at a given time.

The redox state of P700 was determined *in vivo* by using automated routines provided by the Dual-PAM software as described by Schreiber et al.[47]. The P700 signal was determined during a single turnover flashes (ST, 50µs, PQ pools being oxidized) followed by multiple turnover flashes (MT, 50 ms, PQ pools are fully reduced) in the presence of far-red (FR) background light [48], [49]. The complementary area between the oxidation curve of P700 after ST and MT excitation and the stationary level of P700^+^ under FR represents the ST- and MT-areas, respectively. These were used to calculate the functional pools sizes of intersystem electrons relative to P700 as follows: e^−/^P700 = MT-areas/ST-areas [49]. All measurements were conducted at a CO_2_ concentration of approximately 400±10µmol·mol^−1^.

### P515/P535 Measurements

The dual-beam 550 nm to 515 nm difference signal was monitored simultaneously by using the P515/535 module of the Dual-PAM-100 and the automated routines provided by the DUAL-PAM software with minor modifications [50]. After 1 h of dark adaptation, P515 changes induced by saturating single turnover flashes were recorded to evaluate the integrity of the thylakoid membrane. After 10 min of pre-illumination at 531 µmol·m^−2^·s^−1^ and 4 min of dark adaptation, P515 changes induced by saturating single turnover flashes were recorded to evaluate ATPase activity. Slow dark–light–dark induction transients of the 550 nm to 515 nm signals reflect changes in both the membrane potential (electrochromic pigment absorbance shift) and the zeaxanthin content. These transients were measured after 1 h of dark adaptation. Actinic light (AL; 531 µmol·m^−2^·s^−1^) was turned on after 30 s and off after 330 s. Determination of zeaxanthin content, transmembrane potential and proton gradient using the dark-light-dark induction transients was done as described previously by Schreiber et al.[50]. All measurements were performed at a CO_2_ concentration of approximately 400±10µmol·mol^−1^.

### Statistics

Quantitative assessment was conducted on randomly selected samples from four independent biological replicates. Statistical analyses were performed by ANOVA using SPSS version 17.0 (SPSS, Chicago, USA), and comparisons between the mean values were accomplished by the least significant difference test at the 0.05 (or 0.01) probability level. All graphs were made using Origin 8.0 software (Origin Lab, Northampton, MA, USA).

## Results

### Effect of CaCl_2_ on the Net Photosynthetic Rate (Pn) of Tomato Leaves under LNT

**Figure 1:**
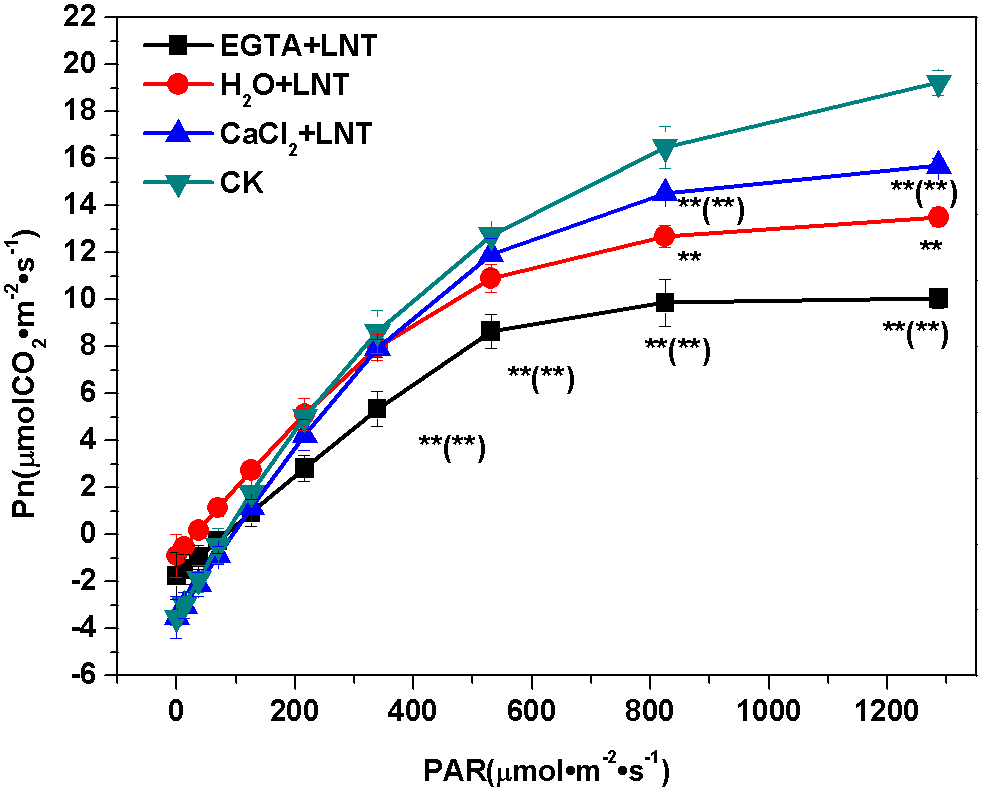
Effect of exogenous calcium pretreatment on the photosynthetic rate of tomato seedlings under LNT. CK, the plant grown at optimal temperature, and used for control; CaCl_2_+LNT, the plant pretreated with CaCl_2_ and grown at low night temperature(LNT); H_2_O+LNT, the plant pretreated with H_2_O and grown at LNT; EGTA+LNT, the plant pretreated with EGTA and grown at LNT. Data are the means of four replicates with standard errors shown by vertical bars. *indicates significant difference (*P*≤0.05), and **indicates a highly significant difference (*P*≤0.01). Comparisons between the figures among all treatments under low night temperature are shown in parentheses.

Compared to the control, the Pn increased slower after LNT treatment and reached a significantly lower value under moderate and high light. In addition, Pn increased markedly with CaCl_2_ pretreatment but decreased with EGTA pretreatment (Fig. 1). These results indicate that the sensitivity of tomato leaves to high light was increased significantly by LNT stress, while CaCl_2_ pretreatment decreased it.

### Effect of CaCl_2_ on the PSII and PSI Activity of Tomato Leaves under LNT

#### Effect of CaCl_2_ on the PSII activity of tomato leaves under LNT

**Figure 2:**
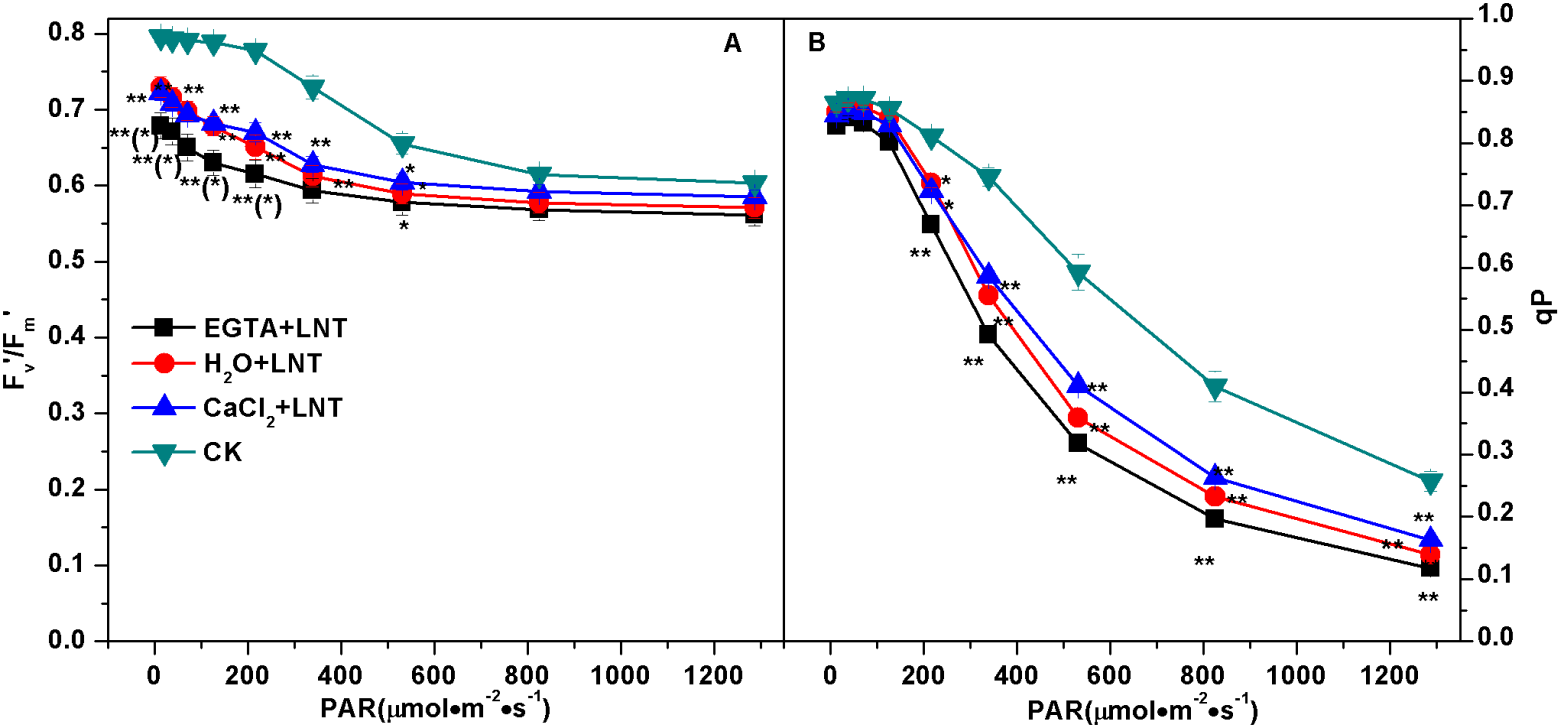
Effect of exogenous calcium pretreatment on the Fv’/Fm’ and qP of tomato seedlings under LNT. Fv’/Fm’, light-adapted maximum quantum yield of PSII; qP, photochemical quenching coefficient. CK, the plant grown at optimal temperature, and used for control; CaCl_2_+LNT, the plant pretreated with CaCl_2_ and grown at low night temperature(LNT); H_2_O+LNT, the plant pretreated with H_2_O and grown at LNT; EGTA+LNT, the plant pretreated with EGTA and grown at LNT. Data are the means of four replicates with standard errors shown by vertical bars. *indicates significant difference (*P*≤0.05), and **indicates a highly significant difference (*P*≤0.01). Comparisons between the figures among all treatments under low night temperature are shown in parentheses.

**Figure 3:**
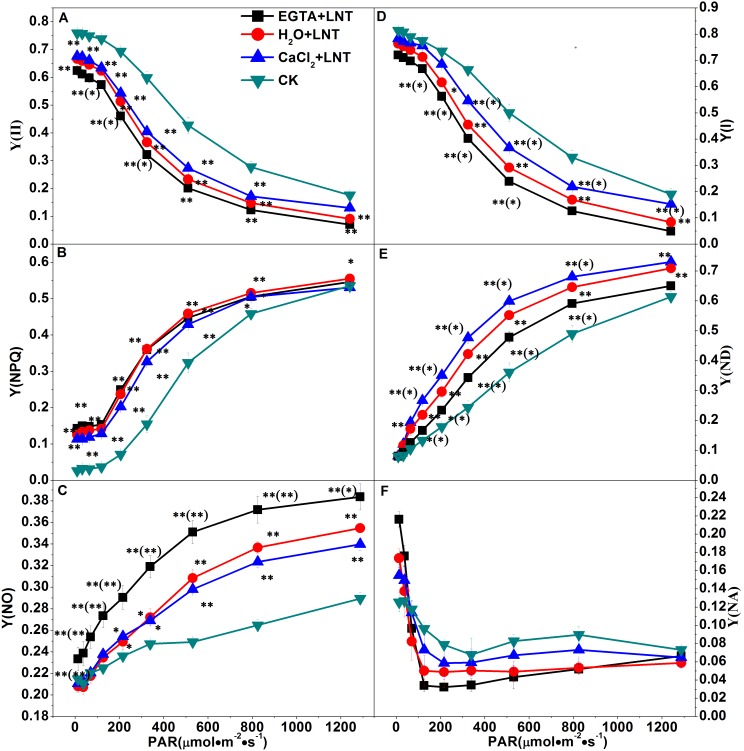
Effect of exogenous calcium pretreatment on energy distribution in photosystems of tomato seedlings under LNT. Y(II), efficient quantum yield of PSII; Y(NPQ), yield of regulated energy dissipation of PSII; Y(NO), yield of non-regulated energy dissipation of PSII; Y(I), quantum yield of PSI; Y(ND), donor side limitation of PSI; Y(NA), acceptor side limitation of PSI. CK, the plant grown at optimal temperature, and used for control; CaCl_2_+LNT, the plant pretreated with CaCl_2_ and grown at low night temperature(LNT); H_2_O+LNT, the plant pretreated with H_2_O and grown at LNT; EGTA+LNT, the plant pretreated with EGTA and grown at LNT. Data are the means of four replicates with standard errors shown by vertical bars. *indicates significant difference (*P*≤0.05), and **indicates a highly significant difference (*P*≤0.01). Comparisons between the figures among all treatments under low night temperature are shown in parentheses.

The Fv’/Fm’ ratio decreased slightly on the rapid light curves across all treatment groups. Compared with the control, Fv’/Fm’ was significantly lower with LNT treatment (Fig. 2A). Meanwhile, CaCl_2_ pretreatment increased it slightly while EGTA pretreatment decreased it. Photochemical quenching (qP) and effective quantum yield of PSII [Y (II)] both showed large decreases at almost all light intensities (Fig. 2B and Fig. 3A, respectively). qP and Y (II) of the LNT treatment group were significantly lower compared to those of the control, especially under high light intensities. CaCl_2_ pretreatment caused a modest elevation in both qP and Y(II), but EGTA pretreatment slightly decreased them. Thus, CaCl_2_ pretreatment and EGTA pretreatment had opposite effects on the PSII activity of tomato leaves under LNT. Since Y(II)] is the product of Fv’/Fm’ multipled by qP, our results indicated that the significant changes in Y(II) was mainly due to the changes in the qP (not the Fv’/Fm’).

The regulatory quantum yield of energy dissipation [Y (NPQ)] and the non-regulatory quantum yield of energy dissipation [Y (NO)] increased gradually with the light intensities (Fig. 3B and Fig. 3C, respectively). Y (NPQ) and Y (NO) after LNT treatment were much higher than those of the control. Compared with the H_2_O and CaCl_2_ pretreatment, the Y (NO) of the EGTA pretreatment was significantly higher. However, the Y (NPQ) showed no difference among the H_2_O pretreatment, CaCl_2_ pretreatment and EGTA pretreatment at all light irradiances (Fig. 3B).

#### Effect of CaCl_2_ on the PSI activity of tomato leaves under LNT

Compared to that of control, the Y (I) of LNT treatment groups were significantly lower (Fig. 3D). Y (ND) increased rapidly with light intensities, and Y(ND) of the LNT treatment group was higher than that of the control at all illumination conditions(Fig. 3E). Y (ND) of EGTA pretreatment was slightly higher than that of the LNT treatment group and was significantly higher than that of the CaCl_2_ pretreatment group. Y(NA) remained high under low irradiance condition (<216µmol·m^−2^·s^−1^), but almost reached stable values under high irradiance condition (531–1287µmol·m^−2^·s^−1^). No significant difference was found among the treatments (Fig. 3F).

### Effect of CaCl_2_ on the Cyclic Electron Transport of Tomato Leaves under LNT

**Figure 4:**
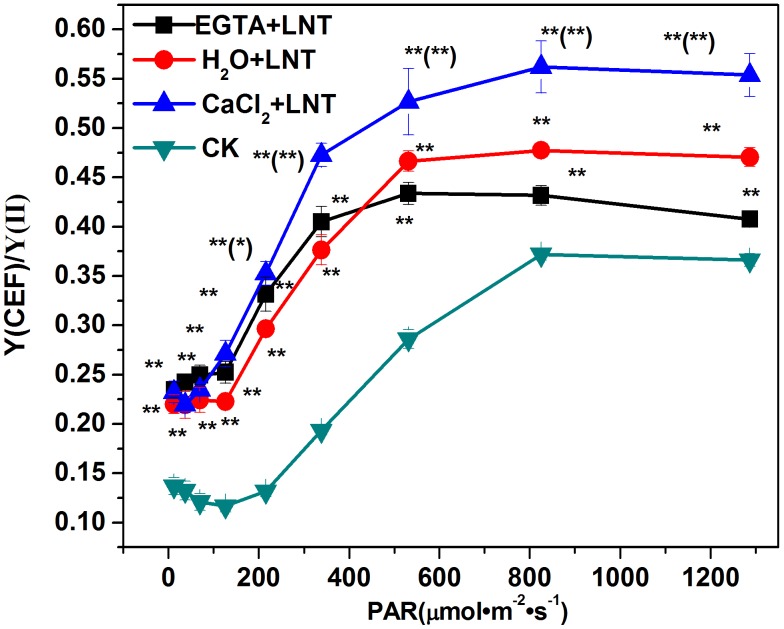
Effect of exogenous calcium pretreatment on the cyclic electron flow of tomato seedlings under LNT. The ratio of the effective quantum yield of cyclic electron flow [Y(CEF)] to the effective quantum yield of PSII [Y(II)] was used to evaluate the operation of cyclic electron flow. CK, the plant grown at optimal temperature, and used for control; CaCl_2_+LNT, the plant pretreated with CaCl_2_ and grown at low night temperature(LNT); H_2_O+LNT, the plant pretreated with H_2_O and grown at LNT; EGTA+LNT, the plant pretreated with EGTA and grown at LNT. Data are the means of four replicates with standard errors shown by vertical bars. *indicates significant difference (P≤0.05), and **indicates a highly significant difference (P≤0.01). Comparisons between the figures among all treatments under low night temperature are shown in parentheses.

Compared to the control, the Y (CEF)/Y (II) ratio of tomato leaves was significantly elevated after LNT treatment. Likewise, the Y (CEF)/Y (II) ratio of tomato leaves increased significantly with the CaCl_2_ pretreatment under LNT, but decreased significantly with EGTA pretreatment (Fig. 4). These results indicate that cyclic electron transport is involved in photo-protection and that exogenous calcium can promote it.

### Effect of CaCl_2_ on the Electron Transport Rate of PSII and PSI of Tomato Leaves under LNT

**Figure 5:**
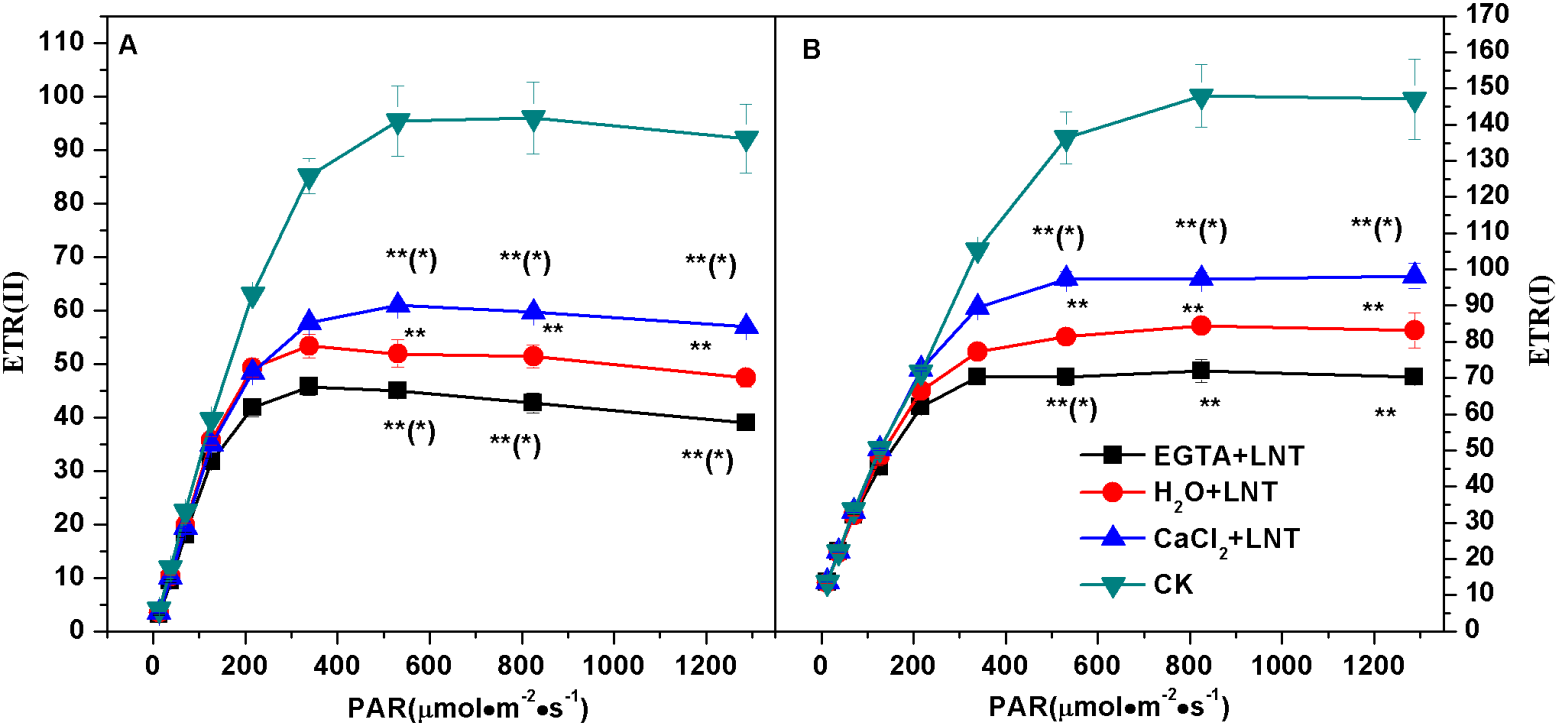
Effect of exogenous calcium pretreatment on electron transport rate tomato seedlings under LNT. ETR(II), electron transport rate of PSII; ETR(I), electron transport rate of PSI. CK, the plant grown at optimal temperature, and used for control; CaCl_2_+LNT, the plant pretreated with CaCl_2_ and grown at low night temperature(LNT); H_2_O+LNT, the plant pretreated with H_2_O and grown at LNT; EGTA+LNT, the plant pretreated with EGTA and grown at LNT. Data are the means of four replicates with standard errors shown by vertical bars. *indicates significant difference (P≤0.05), and **indicates a highly significant difference (P≤0.01). Comparisons between the figures among all treatments under low night temperature are shown in parentheses.

As shown in Figure 5, electron transport rates of both PSII and PSI [ETR (II), ETR (I), respectively] were significantly lower with LNT compared to those of control plants, especially under moderate and high light conditions (Fig. 5A and Fig. 5B, respectively). Exogenous calcium pretreatment improved ETR (II) and ETR (I) significantly, whereas EGTA pretreatment significantly decreased their values under LNT (under moderate and high light conditions). In addition, the higher light level required for the electron transport rate in PSI to reach its maximum value compared to PSII suggests the existence of cyclic electron transport.

### Effect of CaCl_2_ on Fluorescence Rise Kinetics in Saturating Light (O-I_1_-I_2_-P) and PQ Pools of Tomato Leaves under LNT

**Figure 6:**
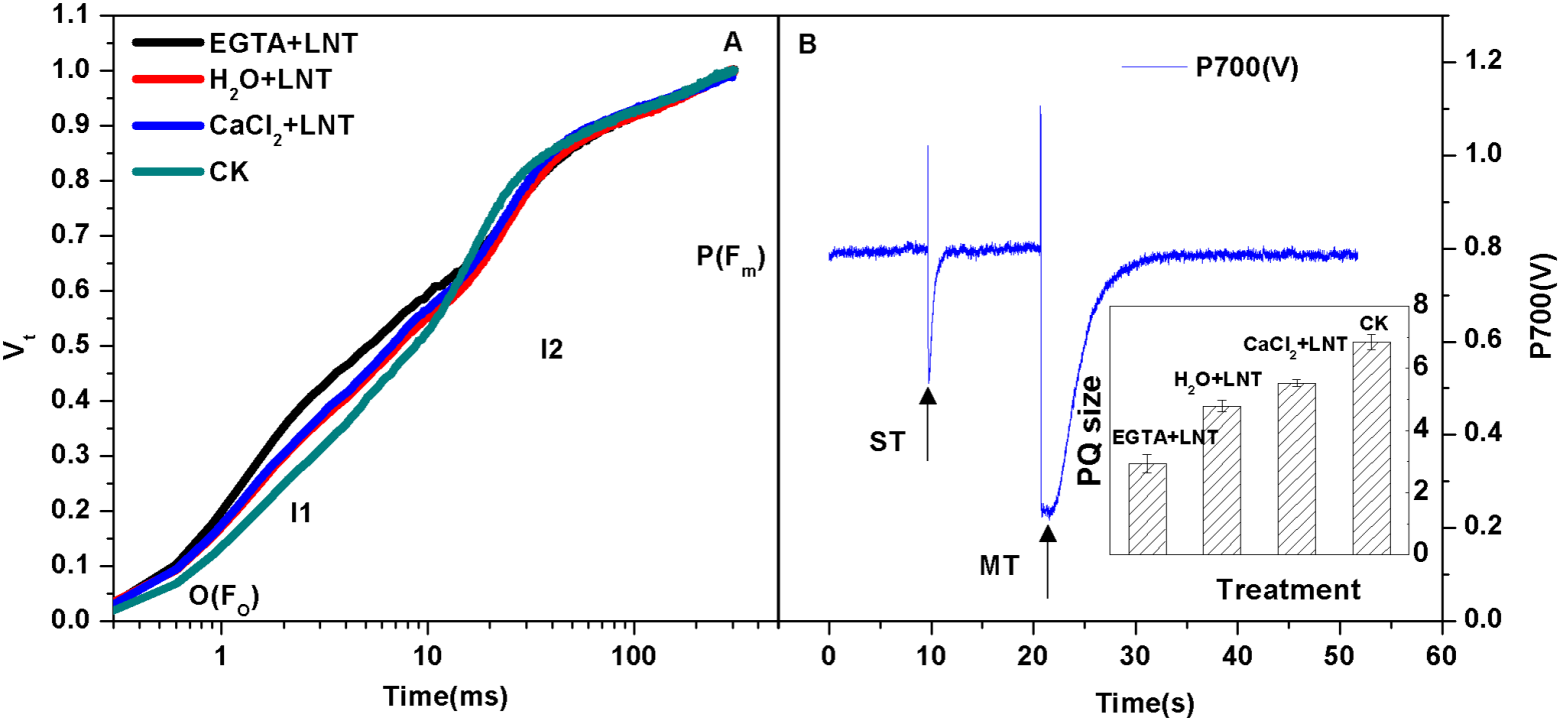
Effect of exogenous calcium pretreatment on the rapid induction kinetics and PQ pools of tomato seedlings under LNT. For Fig. 6A, the rapid induction kinetics upon onset of strong continuous illumination was investigated on the tomato leaves after dark adaptation for 20(Vt) were used to estimate I1-I2 and I2-P contribution and discuss about slow and fast PQ poll reduction. Vt is defined as the ratio of variable fluorescence to the maximal variable fluorescence F_V_ (Fm-Fo), i.e., Vt = (Ft-Fo)/(Fm-Fo), where Ft is the fluorescence at a given time. For Fig. 6B, the P700 signal was determined during a single turnover flashes (ST, 50 µs, PQ pool being oxidized) followed by multiple turnover flashes (MT, 50 ms, PQ pool is fully reduced) in the presence of far-red (FR) background light. The ratio of MT-area/ST-area is used to estimate the size of functional PQ pools. CK, the plant grown at optimal temperature, and used for control; CaCl_2_+LNT, the plant pretreated with CaCl_2_ and grown at low night temperature(LNT); H_2_O+LNT, the plant pretreated with H_2_O and grown at LNT; EGTA+LNT, the plant pretreated with EGTA and grown at LNT. Data are the means of four replicates with standard errors shown by vertical bars. *indicates significant difference (P≤0.05), and **indicates a highly significant difference (P≤0.01). Comparisons between the figures among all treatments under low night temperature are shown in parentheses.

Fig. 6A shows the log time scale of the fast kinetics. The Fo level is seen as a pronounced step. At a given intensity of the saturating light the half-rise time of Fo-I1 (photochemical phase) is about 100 µs. The I1 level is characterized by another pronounced step, followed by the “thermal” I1-I2 and I2-Fm phases. The relative variable fluorescence (Vt) of O-I1, I1-I2, I2-P curves reflect a decrease of Q_A,_ reduction of the fast and slow PQ pools, respectively [46]. Result show that Vt was significantly higher at I1-I2 phases after LNT treatments compared to the control. Furthermore, EGTA pretreatment significantly increased Vt under LNT. However, no difference was observed between LNT treatment and calcium pretreatment groups. Thus, our results show that reduction of fast PQ pools was inhibited after LNT stress, and that EGTA pretreatment aggravated it (Fig. 6A).

In addition, oxidized PQ pools decreased significantly after LNT treatment (Fig. 6B). Compared to the group receiving H_2_O pretreatment under LNT, EGTA pretreatment significantly decreased it, exogenous calcium pretreatment improved it slightly (Fig. 6B).

### Effect of CaCl_2_ on P515 Signal of Tomato Leaves under LNT

**Figure 7:**
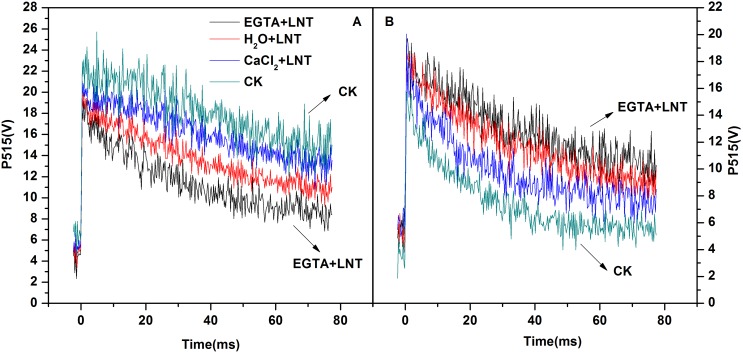
Effect of exogenous calcium pretreatment on the fast P515 signal of tomato seedlings under LNT. (A) for thylakoid membrane, changes in the P515 signal of tomato leaves after 1 h of dark adaptation. (B) for ATPase activity, changes in the P515 signal of tomato leaves after 10 min of pre-illumination at 531 µmol·m^−2^·s^−1^ followed by 4 min of dark adaptation. CK, the plant grown at optimal temperature, and used for control; CaCl_2_+LNT, the plant pretreated with CaCl_2_ and grown at low night temperature(LNT); H_2_O+LNT, the plant pretreated with H_2_O and grown at LNT; EGTA+LNT, the plant pretreated with EGTA and grown at LNT.

Decay of the P515 signal reflects relaxation of the flash induced electric field (created by charge separation in the two photosystems and electrogenic electron transport during Q-cycle in the cyt b/f complex) by H^+^ efflux via the H^+^ channel of the ATPase. A functionally intact photosynthetic apparatus is characterized by slow decay after dark-adaptation (high membrane integrity) and fast decay after illumination (high ATPase activity). Our observations of faster decay after dark adaptation and a slower decay, after illumination with LNT treatment indicated that both the thylakoid membrane was damaged and ATPase activity was reduced after LNT treatment (Fig. 7A and Fig. 7B, respectively). Exogenous calcium pretreatment slightly alleviated the damage of thylakoid membranes and ATPase activity while EGTA pretreatment enhanced the damage caused by LNT stress (Fig. 7A and Fig. 7B, respectively).

**Figure 8:**
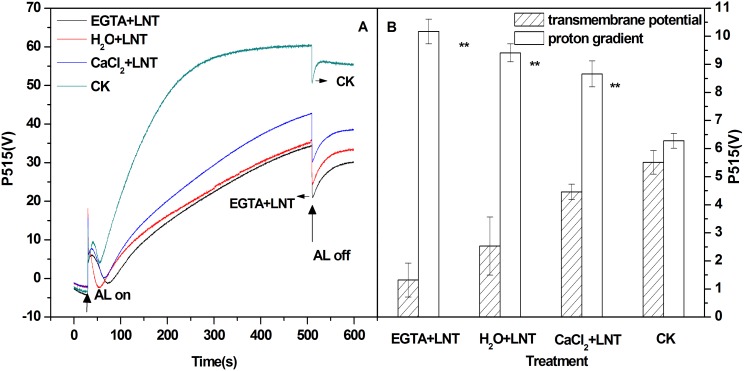
Effect of exogenous calcium pretreatment on slow P515 induction transients of tomato seedlings under LNT. (A) Changes in the P515 signal of slow dark–light–dark induction transients. (B) Two components of the proton motive force (transmembrane potential and proton gradient) derived from the slow dark–light–dark induction transients of the 550 nm to 515 nm signals. The transients were measured after 1 h of dark adaptation. Actinic light (AL; 531 µmol·m^−2^·s^−1^) was turned on after 30 s and off after 330 s. CK, the plant grown at optimal temperature, and used for control; CaCl_2_+LNT, the plant pretreated with CaCl_2_ and grown at low night temperature (LNT); H_2_O+LNT, the plant pretreated with H_2_O and grown at LNT; EGTA+LNT, the plant pretreated with EGTA and grown at LNT. Data are the means of four replicates with standard errors shown by vertical bars. *indicates significant difference (P≤0.05), and **indicates a highly significant difference (P≤0.01). Comparisons between the figures among all treatments under low night temperature are shown in parentheses.

Figure 8A shows dark-light and light-dark induced slow P515 changes in tomato leaves. The observed light-induced signal increase in P515 not only reflects an increase in membrane potential (ECS), but also formation of zeaxanthin [50]. The relative extent of zeaxanthin formation can be judged from the increase in the “dark baseline” apparently after light-off. As showed in Figure 8, the increase in “dark baseline” was significantly lower than that of control after LNT stress indicating decrease in zeaxanthin content. In addition, exogenous calcium pretreatment increased zeaxanthin content while EGTA pretreatment decreased it (Fig. 8A).

The rapid light-off response reflects H^+^ efflux from the lumen to the stroma of chloroplasts via the thylakoid ATPases. The rapid decline in signal decline is followed by a biphasic increase in signal to an apparent “dark baseline.” As indicated in Figure. 8A, the characteristic levels observed during the light-off response can estimate the relative amplitudes of transmembrane potential (Δψ) and ΔpH. The difference between the steady-state signal and the “dark baseline” reflects a substantial Δψ during steady-state illumination. The “undershoot” below the “dark baseline” is considered a measure of the steady-state ΔpH [50]. As shown in Figure. 8B, LNT treatment significantly increased ΔpH and slightly decreased Δψ. Compared to H_2_O pretreatment, exogenous calcium pretreatment decreased ΔpH and slightly increased Δψ, while EGTA pretreatment had opposite effects (Fig. 8B).

## Discussion

### PSII and PSI Performance of Tomato Leaves under LNT

#### PSII performance of tomato leaves under LNT

Our results showed that LNT treatment caused reversible photo-inhibition of PSII (Fig. 3A, Fig. 3C). The decreased capacity for carbon fixation (Fig. 1) and the blocked linear electron transport (Fig. 5) result in potential excess light excitation pressure in PSII reaction centers after LNT treatment. Excess energy in PSII can lead to the generation of reactive oxygen species including ^1^O_2_, O_2_^.-^ and H_2_O_2_ that are deleterious to the electron transport components and protein structure of PSII [1]. Our results showed that Y (NO) was significantly higher than that of the control (Fig. 3C). This suggested that the PSII super-complex may have been damaged and (or) the turnover of D1 may have been disturbed by exposure to excess light energy. Fortunately, plants have developed various photo-protection mechanisms to alleviate the harmful effects of ROS including antioxidant system and NPQ mechanisms. Our previous study showed that the water-water cycle is an effective mechanism to protect the chloroplasts from photodamage under LNT stress [1]. NPQ mechanisms can dissipate the excess energy absorbed by LHCII. Our observation of higher Y(NPQ) after LNT in the present study (Fig. 3B) suggested that although the leaves received excess energy, photochemical energy conversion and protective regulatory mechanisms (such as heat dissipation) was still sufficient to completely consume the light energy absorbed by plants. Since CEF can generate a proton gradient across the thylakoid membrane [22] through transferring electrons from PSI to PQ, it is important for protecting PSII by dissipating excess light energy [20]. CEF can facilitate the NPQ mechanisms including the xanthophyll cycles which require the presence of a proton gradient. Our results showed that the zeaxanthin content was significantly lower after LNT treatment compared to that of the control (Fig. 8A). This indicated the xanthophyll cycles, which is the major mechanism in plants for dissipation of excess energy, was inhibited. In addition, our results showed that stimulation of CEF (not the xanthophyll cycles) was responsible for the high level of Y (NPQ) observed after LNT treatment (Fig. 3B, Fig4, and Fig. 8).

Low temperature is known to play a synergistic role with excess illumination by limiting electron transport and carbon fixation rates. Under these conditions, even a relatively weak light may overwhelm the energy utilization mechanism in plants, resulting in a photoinhibitory effect [51]. In the present study, both carbon fixation capacity and the PSII activity were significantly inhibited under LNT at moderate and high light conditions (Fig. 1 and Fig. 3A, respectively). Compared to the control, Y (NO) under LNT increased more rapidly on the light-response curves. Thus, we conclude that high light condition aggravated the photoinhibition caused by LNT.

Our results showed that the thylakoid membrane integrity and ATP activity were both modestly reduced after LNT treatment (Fig. 7A and Fig. 7B, respectively). Damaged thylakoid membranes may result in decreased capacity of membrane proteins to use light. Stimulation of CEF and reduced ATP activity caused a higher ΔpH across the thylakoid membrane under LNT temperature (Fig. 8B). In addition, the excess higher ΔpH across the thylakoid membrane caused the reduction of zeaxanthin which plays an important role in thermal dissipation in PSII and in scavenging ROS and dissipating singlet Chl [52], [53].

Our results showed that Y (II) was significantly lower with LNT treatment compared to the control. This may be due to inhibition of electron transport between PSII and P700^+^ by LNT (Fig. 5). Since functional PQ pools were significantly inhibited with LNT treatment compared to the control (Fig. 6B), we consider the decrease in PQ as the major factor blocking the electron transport. In addition, the reduction of PQ pools may cause the phosphorylation of thylakoid proteins, activation of state 2 transition to decrease PSII excitation, increase in cyclic electron flow, alleviation of ATP deficit, and increase in proton motive force, thereby down-regulating the PSII antenna via the qE mechanism [54].

#### PSI performance of tomato leaves under LNT

A few studies on PSI have examined isolated thylakoid membranes using artificial electron donors or acceptors [25]. In the present study, we investigated the PSI of tomato plants *in vivo* using a Dual-PAM-100 fluorometer. The absence of changes in Y (NA) compared to the control, indicated that PSI was not inhibited by LNT treatment (Fig. 3F). However, accumulation of NADPH and the over-reduction PSI acceptor contributed to the photo-inhibition of PSI. Generally, a reduced capacity for carbon fixation may cause the over accumulation of NADPH, which can enhance the generation of hydroxyl radicals [55]. Therefore, over-reduced PSI acceptor side can lead to formation of Chl triplets, which can generate the toxic singlet oxygen. Furthermore, the accumulation of NADPH can enhance Mehler reaction, which can generate highly toxic superoxide radicals. Ultimately, hydroxyl radicals, singlet oxygen, and superoxide radicals can cause photoinhibitory damage to PSI. Our studies have indicated that photoinhibitory damage to PSI can be prevented by photo-inhibition of PSII. Furthermore, CEF has an important function in protecting PSI from photo-inhibition. On the one hand, CEF can dissipate excess electron flow to NADPH and O_2_, thereby reducing ROS generation. On the other hand, CEF consumes excess reducing power of NADPH through the NADPH dehydrogenase-dependent pathway [23], [56]. Thus, the observed increase in Y (ND) under LNT was likely caused mainly by the stimulation of CEF (Fig. 3E and Fig. 4, respectively).

### P515 Signals of Tomato Leaves under LNT

The trans-thylakoid proton motive force (pmf), which consists of electrical (Δψ) and osmotic (ΔpH) components, plays two major roles in higher plant photosynthesis. First, both components can drive the synthesis of ATP in the chloroplast ATP synthase. Second, the ΔpH component also plays a key role in regulating photosynthesis, down-regulating the efficiency of light capture by photosynthetic antennae via the qE mechanism which can harmlessly dissipate the excess absorbed light energy as heat [57]. Acidification of the lumen also controls photosynthetic electron transfer by slowing the rate of plastoquinol oxidation at the cytochrome b6f complex, preventing the accumulation of highly reducing species within PSI [58].

Parsing of the thylakoid pmf into ΔpH and Δψ components has been observed in thylakoids and in intact leaves and has been proposed to constitute an important fine-tuning mechanism for photosynthesis [58], [59]. Under normal conditions, a large fraction of pmf can be stored as Δψ, leading to moderate lumen pH and low qE, even at high pmf (and thus high rates of ATP synthesis). In contrast, under environmental stresses–e.g., high light, low CO_2_/O_2_, pmf can be predominantly stored as ΔpH, maximizing lumen acidification for a given pmf [57]–[59]. The partition of pmf into ΔpH and Δψ is affected by three possible factors: capacitance of the thylakoid membrane, ionic composition of the stroma and lumen, and proton-buffering capacity of the lumen [58]. Thus, the ratio ΔpH/ΔΨ can affect the PSII activity and the chemiosmotic ATP synthesis. Our results show that the ratio ΔpH/ΔΨwas significantly higher under LNT and this change was accompanied by lower PSII activity and ATP synthesis compared to the control (Fig. 8B, Fig. 3A and Fig. 7B, respectively). Moreover, respected to lower zeaxanthin content, the higher ΔpH acted qE mechanism predominantly by protonating lumen-exposed residues of PsbS to down-regulating the PSII antenna.

### Effect of Ca^2+^ on the Photoinhibition and Photoprotection of PSII and PSI under LNT

**Figure 9:**
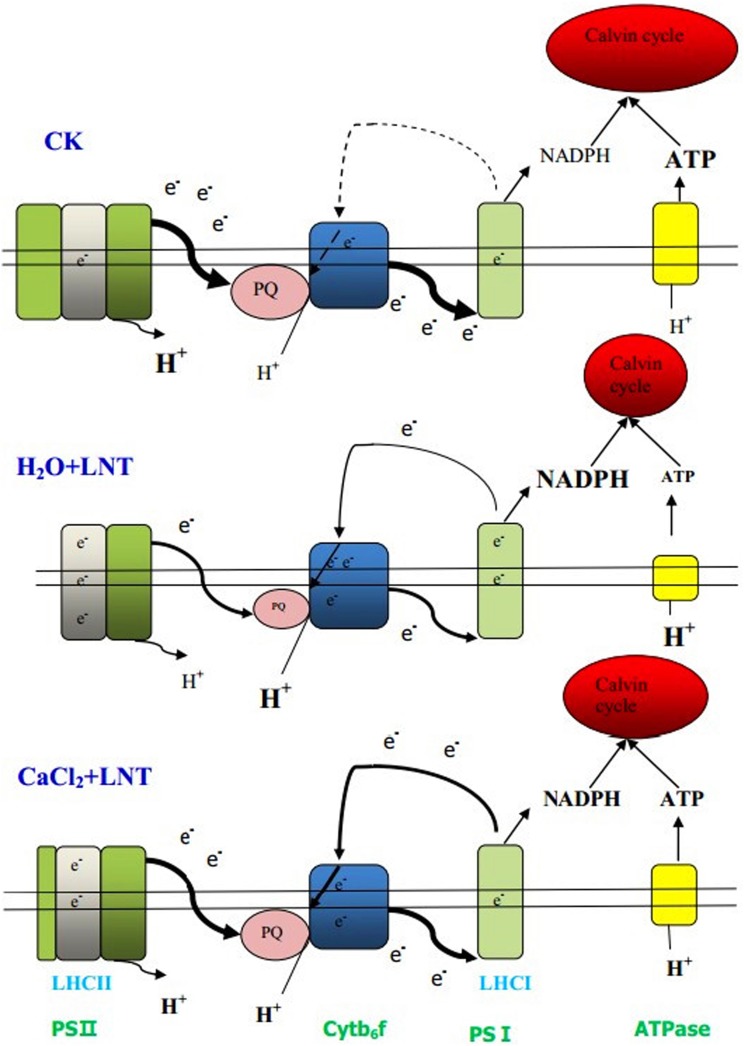
Pictorial representations of thylakoid metabolism changes in the plant pretreated with CaCl_2_ under LNT. The size of units and font represents the activities of the photosynthesis apparatus, and the width of lines represents the extent of electron transport. Exogenous calcium pretreatment can improve decreased linear electron transport, carbon fixation capacity, PQ size, and ATPase activity under low night temperature. Calcium chloride pretreatment can alleviate excess accumulated proton gradient and enhance cyclic electron flow under low night temperature.

Some recent studies have demonstrated that exogenous calcium is effective at improving plant photosynthesis under some conditions of stresses. For example, exogenous calcium was found to be able to mitigate the decline of net photosynthesis rate in peanut under LNT stress [60]. Exogenous calcium improved photosynthesis in heat-stressed tobacco plants [26]. Our results showed that carbon assimilation was improved by the addition of exogenous Ca^2+^ under LNT (Fig. 1). Furthermore, exogenous Ca^2+^ increased oxidized PQ pools under LNT stress (Fig. 6 and Fig. 9). Promotion of carbon fixation and the improvement of PQ pools may be responsible for the higher linear electron transport observed in the CaCl_2_ pretreatment group compared with the H_2_O pretreatment group under LNT (Fig. 5). Previous studies have shown that the calcium-binding protein CAS is crucial for maintaining PSII activity, recovery, and/or turnover, as well as in driving high light acclimation [36], [61]. In the present study, exogenous calcium only slightly decreased Y (NO), whereas EGTA pretreatment significantly increased it under LNT (Fig. 3C). Thus, PSII reaction centers were severely damaged. The slightly acidic pH (Fig. 8) driven by CEF (Fig. 4) with calcium pretreatment group possibly promoted the binding of calcium to PsbO, which is important in the assembly and stabilization of PSII reaction centers [54]. Moreover, Ca^2+^ can affect the expression of LHC stress-related protein 3, which is crucial for qE, the energy-dependent component of NPQ [37]. However, CEF contributes to the pH gradient across the thylakoid membrane, which is required for efficient qE. Application of Ca^2+^ may also increase the binding of CaM to NADK2, which is known to modulate the NAD/NADP balance [36], [61]. Furthermore, our study also showed that ATPase activity was promoted by exogenous calcium under LNT (Fig. 7B). This result is in line with a previous study on tobacco under high temperature stress [26].

In the present study, we have found that EGTA can significantly reduce carbon fixation (Fig. 1). EGTA has been shown previously to exacerbate the decline of photosynthesis due to stomatal limitation under LNT stress [60]. In addition, we found that EGTA aggravated the photoinhibiton caused by LNT stress, as revealed by significantly decreased Y(II) and significantly increased Y(NO). This may have been caused mainly by inhibition of the electron transport rate and the stimulation of the CEF and xanthophyll cycles (Fig. 4, Fig. 5 and Fig. 8, respectively). This is also consistent with our result that application of Ca^2+^ could alleviate the photoinhibition caused by LNT stress.

In summary, we found that exogenous Ca^2+^ facilitated the photo-protection mechanisms, including the CEF and the xanthophyll cycles, under LNT stress. We propose that enhanced photo-protection and greater stabilization of the photosynthetic apparatus together underlie the mechanism by which exogenous Ca^2+^ alleviates the photo-inhibition caused by LNT stress (Fig. 9).

## Conclusion

In conclusion, the selective photo-inhibition of PSII observed after LNT stress can be attributed to decreased carbon fixation, blocked linear electron transport caused by the inhibition of fast PQ pools reduction and the decrease of oxidized PQ pools, and closed PSII reaction centers. CEF can protect PSII and PSI from LNT-induced photo-inhibition. Exogenous Ca^2+^ alleviates PSII photo-inhibition caused by LNT stress mainly by promoting carbon fixation, CEF, xanthophyll cycles, PQ pools, and ATPase activity.

## References

[1] Liu YF, Qi MF, Li TL (2012) Photosynthesis, photoinhibition, and antioxidant system in tomato leaves stressed by low night temperature and their subsequent recovery. Plant Science 196: 8–17. 23017895 10.1016/j.plantsci.2012.07.005

[2] Liu Y, Li T, Xu T, Qi M, Xu C, et al (2011) Effect of low night temperature treatment and recovery on photosynthesis and the allocation of absorbed light energy in tomato (Lycopersicon esculentum Mill.) leaves. Journal of Horticultural Science and Biotechnology 86: 91.

[3] Powles SB (1984) Photoinhibition of photosynthesis induced by visible light. Annual Review of Plant Physiology 35: 15–44.

[4] De Las Rivas J, Andersson B, Barber J (1992) Two sites of primary degradation of the D1-protein induced by acceptor or donor side photo-inhibition in photosystem II core complexes. FEBS Letters 301: 246–252. 1577160 10.1016/0014-5793(92)80250-k

[5] Huang W, Zhang S-B, Cao K-F (2010) The different effects of chilling stress under moderate light intensity on photosystem II compared with photosystem I and subsequent recovery in tropical tree species. Photosynth Res 103: 175–182. 20221850 10.1007/s11120-010-9539-7

[6] Zhang S, Scheller HV (2004) Photoinhibition of photosystem I at chilling temperature and subsequent recovery in Arabidopsis thaliana. Plant and Cell Physiology 45: 1595–1602. 15574835 10.1093/pcp/pch180

[7] Havaux M, Greppin H, Strasser R (1991) Functioning of photosystems I and II in pea leaves exposed to heat stress in the presence or absence of light. Planta 186: 88–98. 24186579 10.1007/BF00201502

[8] Dongsansuk A, Lütz C, Neuner G (2013) Effects of temperature and irradiance on quantum yield of PSII photochemistry and xanthophyll cycles in a tropical and a temperate species. Photosynthetica 51: 13–21.

[9] Ivanov AG, Allakhverdiev SI, Huner NPA, Murata N (2012) Genetic decrease in fatty acid unsaturation of phosphatidylglycerol increased photoinhibition of photosystem I at low temperature in tobacco leaves. Biochimica et Biophysica Acta (BBA) - Bioenergetics 1817: 1374–1379. 22445720 10.1016/j.bbabio.2012.03.010

[10] Scheller H, Haldrup A (2005) Photoinhibition of photosystem I. Planta. 221: 5–8. 10.1007/s00425-005-1507-7 15782347

[11] Sonoike K, Terashima I (1994) Mechanism of photosystem-I photoinhibition in leaves of Cucumis sativus L. Planta. 194: 287–293.

[12] Pospíšil P (2012) Molecular mechanisms of production and scavenging of reactive oxygen species by photosystem II. Biochimica et Biophysica Acta (BBA) - Bioenergetics 1817: 218–231. 21641332 10.1016/j.bbabio.2011.05.017

[13] Chen L, Jia H, Tian Q, Du L, Gao Y, et al (2012) Protecting effect of phosphorylation on oxidative damage of D1 protein by down-regulating the production of superoxide anion in photosystem II membranes under high light. Photosynth Res 112: 141–148. 22644478 10.1007/s11120-012-9750-9

[14] Hideg É, Ogawa Ki, Kálai T, Hideg K (2001) Singlet oxygen imaging in Arabidopsis thaliana leaves under photoinhibition by excess photosynthetically active radiation. Physiologia Plantarum 112: 10–14. 11319009 10.1034/j.1399-3054.2001.1120102.x

[15] Miyao M, Ikeuchi M, Yamamoto N, Ono T-a (1995) Specific degradation of the D1 protein of photosystem II by treatment with hydrogen peroxide in darkness: implications for the mechanism of degradation of the D1 protein under illumination. Biochemistry 34: 10019–10026. 7632674 10.1021/bi00031a025

[16] Partelli FL, Batista-Santos P, Scotti-Campos P, Pais IP, Quartin VL, et al (2011) Characterization of the main lipid components of chloroplast membranes and cold induced changes in Coffea spp. Environmental and Experimental Botany 74: 194–204.

[17] Kanazawa A, Kramer DM (2002) In vivo modulation of nonphotochemical exciton quenching (NPQ) by regulation of the chloroplast ATP synthase. Proceedings of the National Academy of Sciences 99: 12789–12794. 10.1073/pnas.182427499 PMC130538 12192092

[18] Joliot P, Alric J (2013) Inhibition of CO2 fixation by iodoacetamide stimulates cyclic electron flow and non-photochemical quenching upon far-red illumination. Photosynth Res 115: 55–63. 23625532 10.1007/s11120-013-9826-1

[19] Miyake C, Shinzaki Y, Miyata M, Tomizawa K-i (2004) Enhancement of cyclic electron flow around PSI at high light and its contribution to the induction of non-photochemical quenching of Chl fuorescence in intact leaves of tobacco plants. Plant and Cell Physiology 45: 1426–1433. 15564526 10.1093/pcp/pch163

[20] Takahashi S, Milward SE, Fan D-Y, Chow WS, Badger MR (2009) How does cyclic electron flow alleviate photoinhibition in Arabidopsis? Plant Physiology 149: 1560–1567. 19118124 10.1104/pp.108.134122 PMC2649389

[21] Shikanai T (2014) Central role of cyclic electron transport around photosystem I in the regulation of photosynthesis. Current Opinion in Biotechnology 26: 25–30. 24679254 10.1016/j.copbio.2013.08.012

[22] Munekage Y, Hojo M, Meurer J, Endo T, Tasaka M, et al (2002) PGR5 is involved in cyclic electron flow around photosystem I and is essential for photoprotection in Arabidopsis. Cell 110: 361–371. 12176323 10.1016/s0092-8674(02)00867-x

[23] Shikanai T (2007) Cyclic electron transport around photosystem I: Genetic Approaches. Annual Review of Plant Biology 58: 199–217. 10.1146/annurev.arplant.58.091406.110525 17201689

[24] Chen H-X, Li P-M, Gao H-Y (2007) Alleviation of photoinhibition by calcium supplement in salt-treated Rumex leaves. Physiologia Plantarum 129: 386–396.

[25] Huang J, Zhang J, Yu YM, Liu JX, Li MJ, et al (2012) Effects of calcium fertilizer on the development, survival, and feeding of B-biotype Bemisia tabaci on Euphorbia pulcherrima. The journal of applied ecology 23: 2521–2528. 23286011

[26] Tan W, Meng Qw, Brestic M, Olsovska K, Yang X (2011) Photosynthesis is improved by exogenous calcium in heat-stressed tobacco plants. Journal of Plant Physiology 168: 2063–2071. 21803445 10.1016/j.jplph.2011.06.009

[27] Kader MA, Lindberg S (2010) Cytosolic calcium and pH signaling in plants under salinity stress. Plant Signaling & Behavior 5: 233–238. 20037468 10.4161/psb.5.3.10740 PMC2881266

[28] Qi MF, Liu YF, Zhou LF, Li TL, Fan Y, H, et al. (2011) Regulation of calcium on photosynthesis of tomato leaves under sub-high temperature stress. Scientia Agricultura Sinica. 531–537.

[29] Xu C, Li X, Zhang L (2013) The effect of calcium chloride on growth, photosynthesis, and antioxidant responses of Zoysia japonica under drought conditions. PloS one 8: e68214. 23844172 10.1371/journal.pone.0068214 PMC3699550

[30] Agarwal S, Sairam RK, Srivastava GC, Tyagi A, Meena RC (2005) Role of ABA, salicylic acid, calcium and hydrogen peroxide on antioxidant enzymes induction in wheat seedlings. Plant Science 169: 559–570.

[31] Ai XZ, Wang XF, Guo YK, Xing YX (2006) Effects of suboptimal temperature and low temperature under low light intensity on stomatal characteristics and chloroplast ultrastructure of cucumber seedlings. Scientia Agricultura Sinica 39: 2063–2068.

[32] You JH, Lu JM, Yang WJ (2002) Effects of Ca^2+^ on photosynthesis and related physiological indexes of wheat seedlings under low temperature stress. Acta Agronomica Sinica 28: 693–696.

[33] Bayer RG, Stael S, Rocha AG, Mair A, Vothknecht UC, et al (2012) Chloroplast-localized protein kinases: a step forward towards a complete inventory. Journal of Experimental Botany 63: 1713–1723. 22282538 10.1093/jxb/err377 PMC3971369

[34] Chigri F, Hörmann F, Stamp A, Stammers DK, Bölter B, et al (2006) Calcium regulation of chloroplast protein translocation is mediated by calmodulin binding to Tic32. Proceedings of the National Academy of Sciences 103: 16051–16056. 10.1073/pnas.0607150103 PMC1635125 17035502

[35] Stael S, Bayer RG, Mehlmer N, Teige M (2011) Protein N-acylation overrides differing targeting signals. FEBS Letters 585: 517–522. 21219905 10.1016/j.febslet.2011.01.001 PMC3971372

[36] Turner WL, Waller JC, Vanderbeld B, Snedden WA (2004) Cloning and characterization of two NAD kinases from Arabidopsis. Identification of a calmodulin binding isoform. Plant Physiology 135: 1243–1255. 15247403 10.1104/pp.104.040428 PMC519044

[37] Terashima M, Petroutsos D, Hüdig M, Tolstygina I, Trompelt K, et al (2012) Calcium-dependent regulation of cyclic photosynthetic electron transfer by a CAS, ANR1, and PGRL1 complex. Proceedings of the National Academy of Sciences 109: 17717–17722. 10.1073/pnas.1207118109 PMC3491457 23045639

[38] Feng Z, Liang F, Zheng C-s, Shu H-r, Sun X-z, et al (2010) Effects of acetylsalicylic acid and calcium chloride on photosynthetic apparatus and reactive oxygen-scavenging enzymes in chrysanthemum Under low temperature stress with low Light. Agricultural Sciences in China 9: 1777–1786.

[39] Bellamine J, Greppin H (1997) Involvement of calcium in the in vitro sensitivity change of the plasma membrane H+ ATPase to indole-3-acetic acid. Acta Physiologiae Plantarum 19: 17–22.

[40] Yamori W, Sakata N, Suzuki Y, Shikanai T, Makino A (2011) Cyclic electron flow around photosystem I via chloroplast NAD(P)H dehydrogenase (NDH) complex performs a significant physiological role during photosynthesis and plant growth at low temperature in rice. The Plant Journal 68: 966–976. 21848656 10.1111/j.1365-313X.2011.04747.x

[41] Genty B, Briantais J-M, Baker NR (1989) The relationship between the quantum yield of photosynthetic electron transport and quenching of chlorophyll fluorescence. Biochimica et Biophysica Acta (BBA) - General Subjects 990: 87–92.

[42] Kramer D, Johnson G, Kiirats O, Edwards G (2004) New fluorescence parameters for the determination of QA redox state and excitation energy fluxes. Photosynth Res 79: 209–218. 16228395 10.1023/B:PRES.0000015391.99477.0d

[43] Busch F, Hüner NPA, Ensminger I (2009) Biochemical constrains limit the potential of the photochemical reflectance index as a predictor of effective quantum efficiency of photosynthesis during the winter spring transition in Jack pine seedlings. Functional Plant Biology 36: 1016–1026. 10.1071/FP08043 32688713

[44] Klughammer C, Schreiber U (1994) An improved method, using saturating light pulses, for the determination of photosystem I quantum yield via P700^+^-absorbance changes at 830 nm. Planta 192: 261–268.

[45] Schreiber U, Hormann H, Neubauer C, Klughammer C (1995) Assessment of photosystem II photochemical quantum yield by chlorophyll fluorescence quenching analysis. Functional Plant Biology 22: 209–220.

[46] Lazár D (2006) The polyphasic chlorophyll a fluorescence rise measured under high intensity of exciting light. Functional Plant Biology 33: 9. 10.1071/FP05095 32689211

[47] Schreiber U (1988) Measuring P700 absorbance changes around 830 nm with a new type of pulse modulation system. Z Naturforsch 43: 686–698.

[48] Schreiber U, Neubauer C, Klughammer C (1989) Devices and Methods for Room-Temperature Fluorescence Analysis. Philosophical Transactions of the Royal Society B: Biological Sciences 323: 241–251.

[49] Savitch LV, Barker-Åstrom J, Ivanov AG, Hurry V, Öquist G, et al (2001) Cold acclimation of Arabidopsis thaliana results in incomplete recovery of photosynthetic capacity, associated with an increased reduction of the chloroplast stroma. Planta 214: 295–303. 11800395 10.1007/s004250100622

[50] Schreiber U, Klughammer C (2008) New accessory for the Dual-PAM-100: The P515/535 module and examples of its application. PAN 1: 1–10.

[51] Gerotto C, Alboresi A, Giacometti GM, Bassi R, Morosinotto T (2011) Role of PSBS and LHCSR in Physcomitrella patens acclimation to high light and low temperature. Plant, Cell & Environment 34: 922–932. 10.1111/j.1365-3040.2011.02294.x 21332514

[52] Havaux M, Niyogi KK (1999) The violaxanthin cycle protects plants from photooxidative damage by more than one mechanism. Proceedings of the National Academy of Sciences 96: 8762–8767. 10.1073/pnas.96.15.8762 PMC17590 10411949

[53] Müller P, Li X-P, Niyogi KK (2001) Non-photochemical quenching. A response to excess light energy. Plant Physiology 125: 1558–1566. 11299337 10.1104/pp.125.4.1558 PMC1539381

[54] Yi X, McChargue M, Laborde S, Frankel LK, Bricker TM (2005) The manganese-stabilizing protein is required for photosystem II assembly/stability and photoautotrophy in higher plants. Journal of Biological Chemistry 280: 16170–16174. 15722336 10.1074/jbc.M501550200

[55] Mi H, Klughammer C, Schreiber U (2000) Light-induced dynamic changes of NADPH fluorescence in Synechocystis PCC 6803 and its ndhB-defective mutant M55. Plant and Cell Physiology 41: 1129–1135. 11148271 10.1093/pcp/pcd038

[56] Huang W, Zhang S-B, Cao K-F (2011) Cyclic Electron Flow Plays an Important Role in Photoprotection of Tropical Trees Illuminated at Temporal Chilling Temperature. Plant and Cell Physiology 52: 297–305. 21062868 10.1093/pcp/pcq166

[57] Ioannidis NE, Cruz JA, Kotzabasis K, Kramer DM (2012) Evidence that putrescine modulates the higher plant photosynthetic proton circuit. PloS one 7: e29864. 22253808 10.1371/journal.pone.0029864 PMC3257247

[58] Ioannidis NE, Kotzabasis K (2014) Polyamines in chemiosmosis in vivo: A cunning mechanism for the regulation of ATP synthesis during growth and stress. Frontiers in plant science 5. 10.3389/fpls.2014.00071 PMC3938100 24592272

[59] Avenson TJ, Cruz JA, Kanazawa A, Kramer DM (2005) Regulating the proton budget of higher plant photosynthesis. Proceedings of the National Academy of Sciences of the United States of America 102: 9709–9713. 15972806 10.1073/pnas.0503952102 PMC1172270

[60] Liu Y-f, Han X-r, Zhan X-m, Yang J-f, Wang Y-z, et al (2013) Regulation of Calcium on Peanut Photosynthesis Under Low Night Temperature Stress. Journal of Integrative Agriculture 12: 2172–2178.

[61] Rocha A, Vothknecht U (2012) The role of calcium in chloroplasts–an intriguing and unresolved puzzle. Protoplasma 249: 957–966. 22227834 10.1007/s00709-011-0373-3

